# Supplementary material for: The Kinetics of Aragonite Formation from Solution via Amorphous Calcium Carbonate
Source: Nanomaterials (Basel). 2022 Nov 23;12(23):4151. doi: 10.3390/nano12234151 (PMC9739954; doi:10.3390/nano12234151)
Supplement: Supplementary file 1 [file nanomaterials-12-04151-s001.zip › nanomaterials-1998719-supplementary.pdf]

Supplementary Materials

The Kinetics of Aragonite Formation from Solution via Amorphous Calcium Carbonate

Simon M. Clark [1,2,*], Vili Grigorova [1], Bruno Colas [1,2], Tamim A. Darwish [3], Kathleen Wood [2], Joerg Neuefeind [4] and Dorrit E. Jacob [5]

1 School of Engineering, Faculty of Science and Engineering, Macquarie University, North Macquarie Park, NSW 2109, Australia
2 Australian Centre for Neutron Scattering, Australian Nuclear Science and Technology Organisation, Locked Bag 2001, Kirrawee DC, NSW 2232, Australia
3 National Deuteration Facility, Australian Nuclear Science and Technology Organisation, Kirrawee, DC, Australia
4 Spallation Neutron Source, Oak Ridge National Laboratory, Oak Ridge, TN 37831, USA
5 Research School of Earth Sciences, The Australian National University, Canberra, ACT 2600, Australia
* Correspondence: simon.clark@mq.edu.au

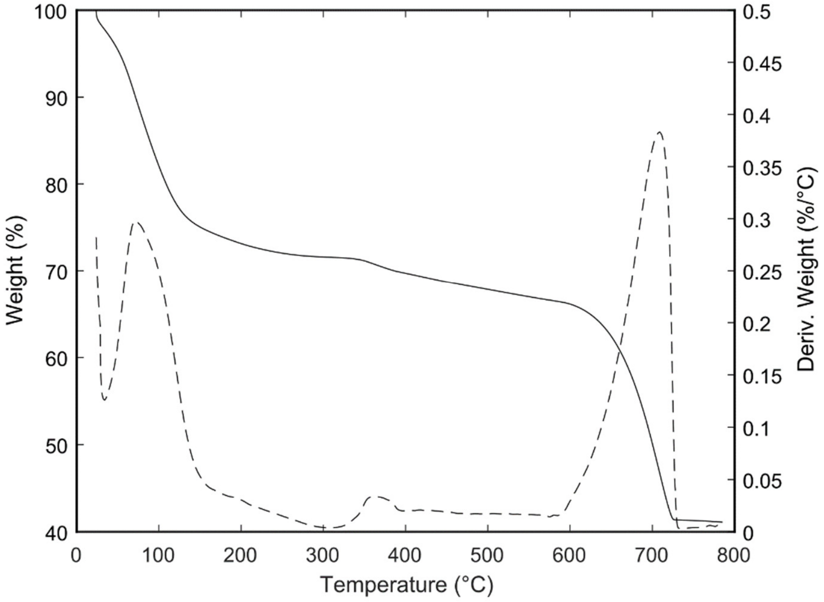

Figure S1: Typical TGA pattern of an ACC sample, with the derivative weight shown by a dashed line. The derivative weight is plotted to highlight the weight change.

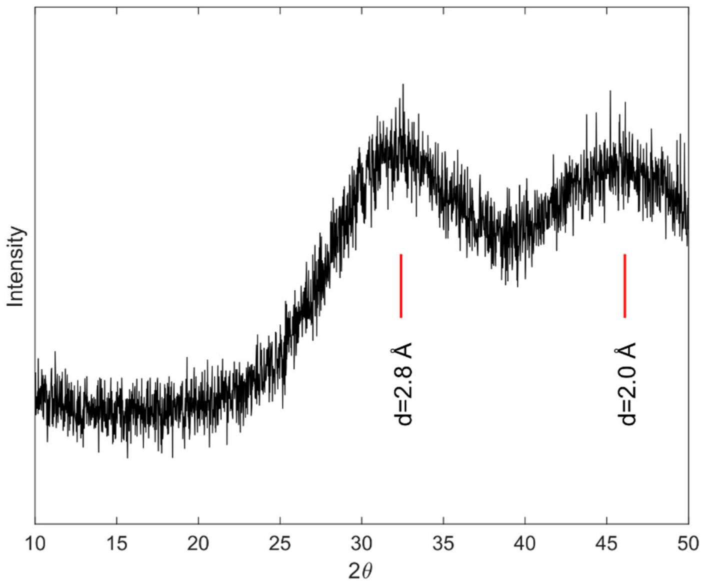

Figure S2: X-ray powder diffraction pattern of our Mg-ACC sample with two diffuse maxima at d=2.8 Å and d=2.0 Å and displaying no sharp diffraction peaks.

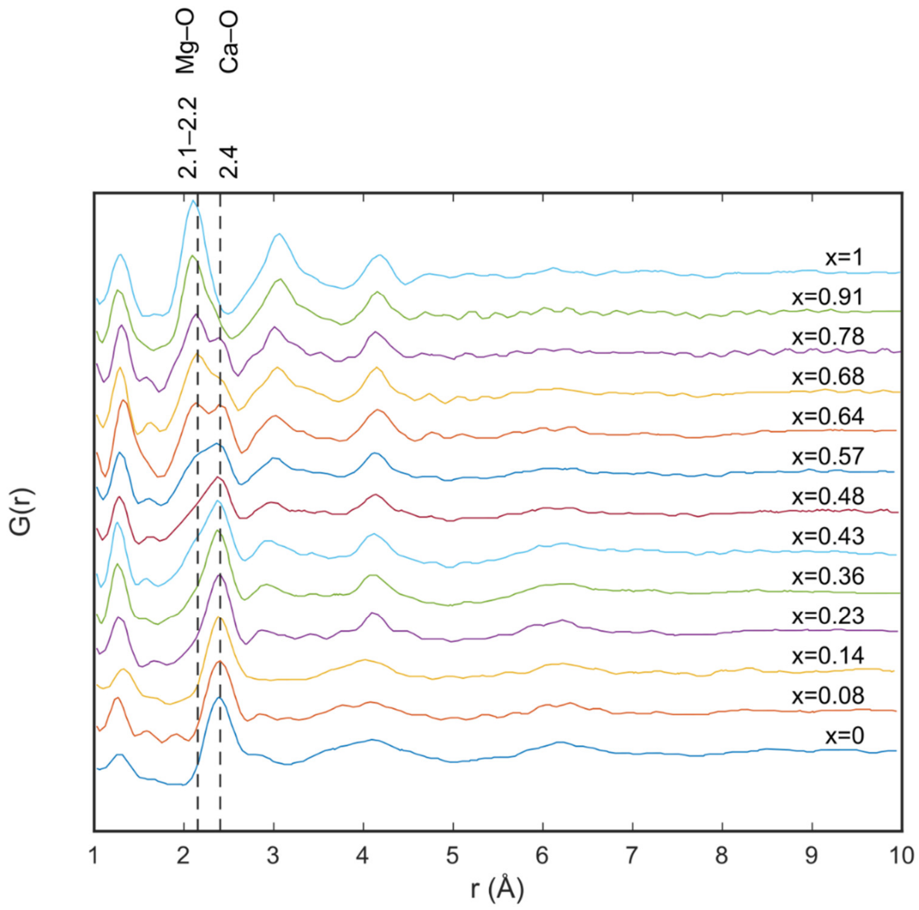

Figure S3. X-ray scattering of solid-solution mixtures of $(Ca_{1-x}Mg_x)CO_3$ [31]. With x=0 for amorphous calcium carbonate (ACC) and x=1 for amorphous magnesium carbonate (AMC). The two dashed lines around 2.1—2.2 Å and 2.4 Å represent respectively the first Mg-O and Ca-O correlations.

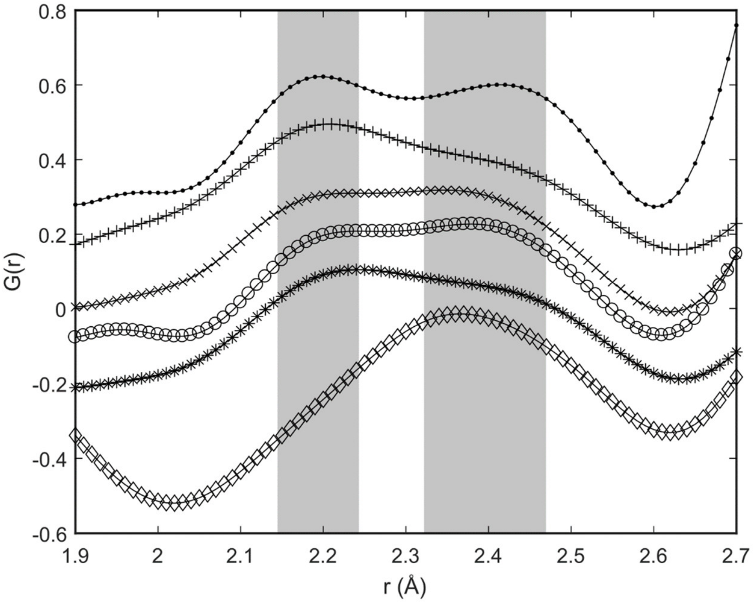

Figure S4. Neutron scattering patterns of samples prepared with different Mg/Ca ratios: —•— x=0.39, —+— x=0.26, —×— x=0.2, —○— x=0.13, —∗— x=0.07, and —◇— x=0; taken from $(Ca_{1-x}\ Mg_x)CO_3$ mixtures. The two grey bands around 2.2 Å and 2.4 Å represent, similarly that on **Error! Reference source not found.**, respectively the first Mg-O and Ca-O correlations.

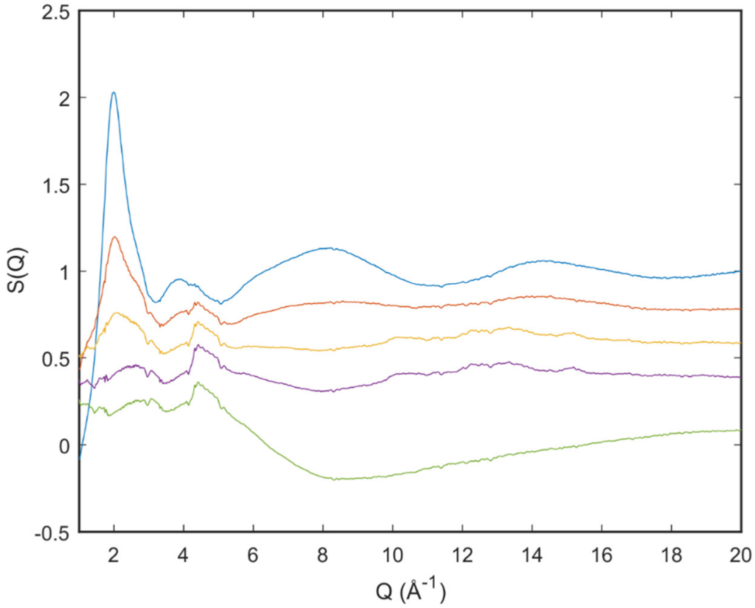

Figure S5. Neutron scattering data of H_2O / D_2O mixtures consisting of, from top to bottom: 100% H_2O, 60% H_2O – 40% D_2O, 40\% H_2O – 60% D_2O, 20% H_2O – 80% D_2O, 100% D_2O.

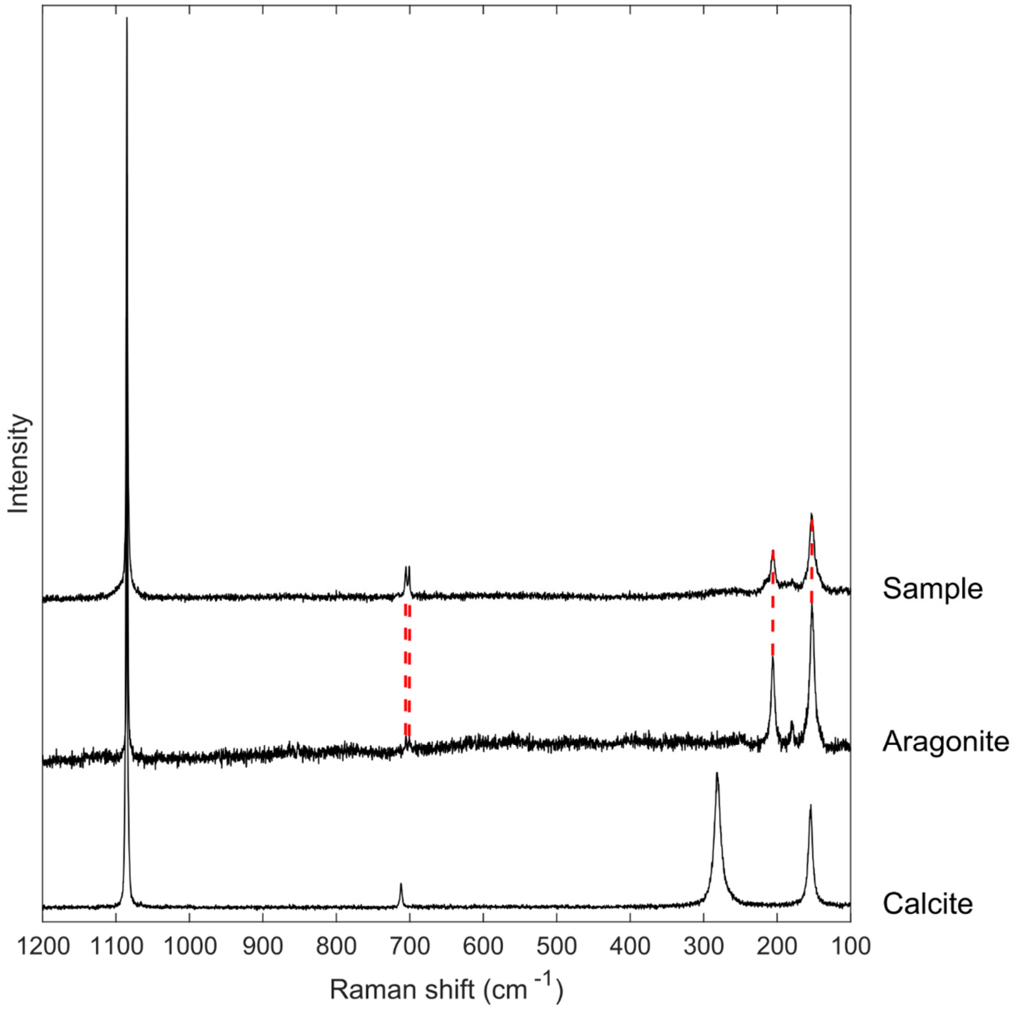

Figure S6. Representative Raman spectrum collected from a crystallised ACC sample (top) compared to spectra collected from an aragonite (middle) and a calcite (bottom) sample demonstrating that our ACC samples give only aragonite on crystallisation.

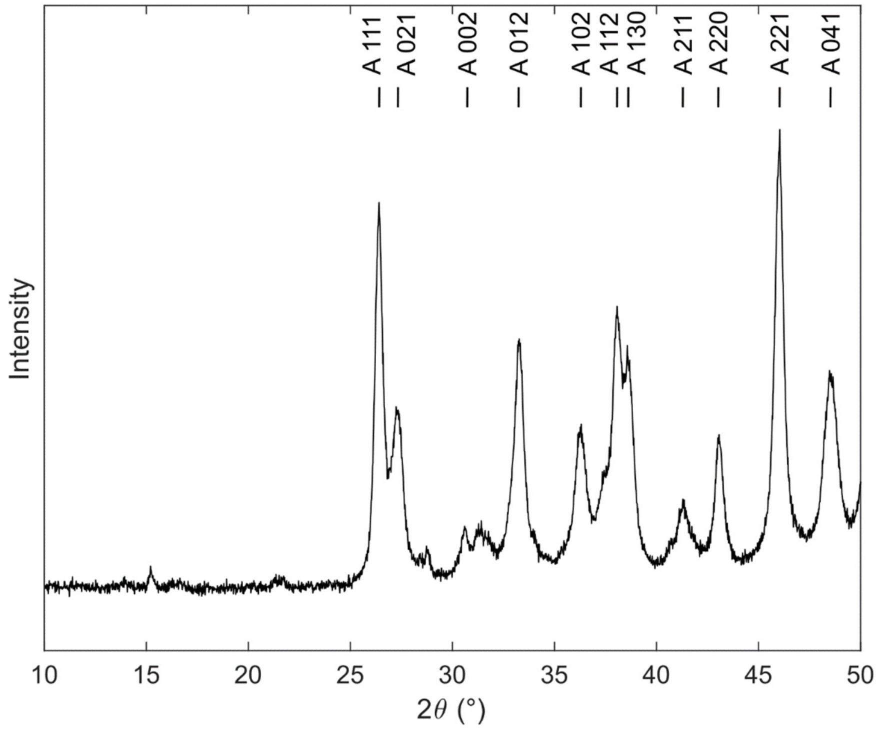

Figure S7. X-ray powder diffraction pattern of an ACC converted into aragonite. Peak assignations in the format A for aragonite followed by the miller indices hkl.

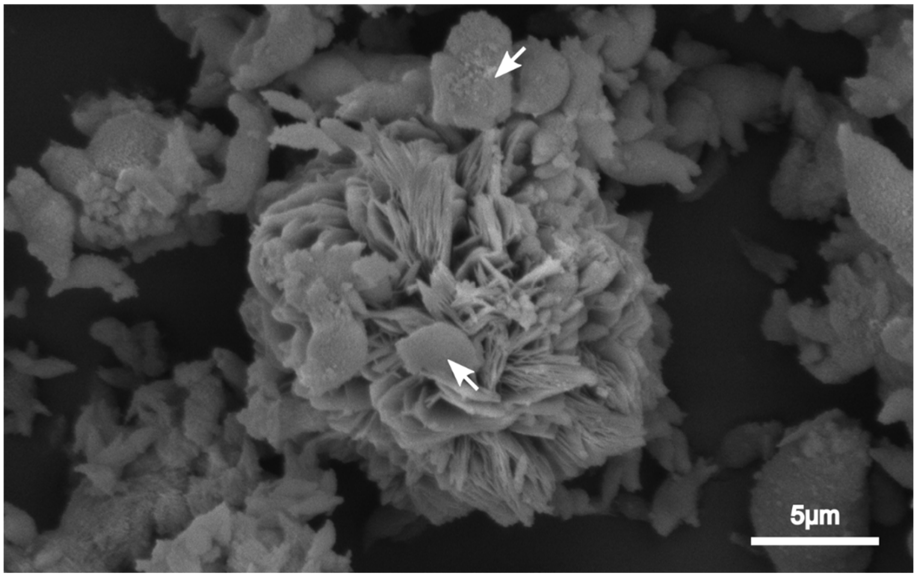

Figure S8. SEM micrograph of a synthesised sample from this study showing tablets of aragonite and xenomorphic aggregates believed to be ACC (two of them are pointed with white arrows), similar to such kind of aragonite seen in nacre [38].

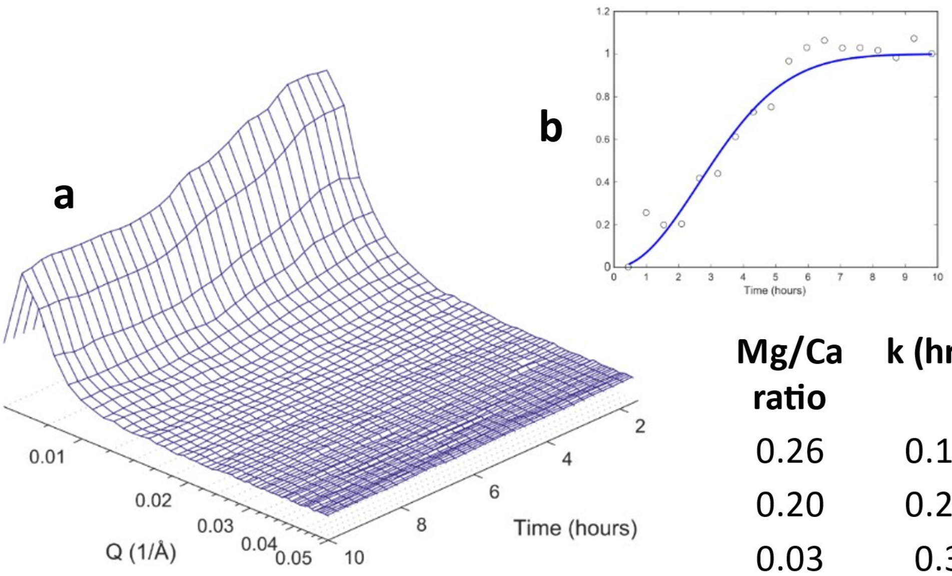

Mg/Ca ratio	k (hr^{-1})
0.26	0.17
0.20	0.27
0.03	0.3

Figure S9. **a.** SANS patterns evolving with time of the sample with Mg/Ca ratio = 0.20 at 20°C. **b.** Open circles show the evolution of the peak height for this sample. The blue line is the fit of the data using an Avrami equation $y = 1 - e^{-(kt)^2}$, with $k = 0.27$. The table contains values for the rate of formation of the ACC particles for three Mg/Ca ratios.

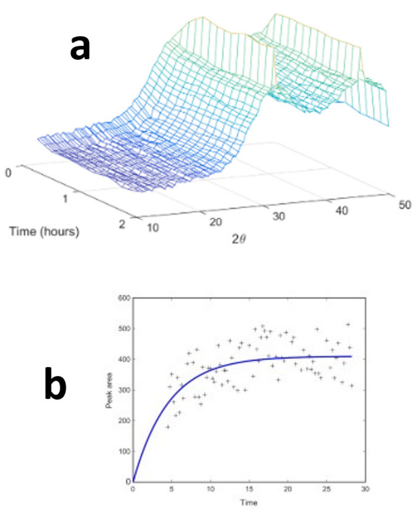

Mg/Ca ratio	k (H) (h^{-1})	k (D) (h^{-1})
0.096	1.08	
0.116	0.17	
0.117	0.17	
0.137		0.02
0.133	0.1	
0.110	0.88	
0.116		0.07
0.120		0.02
0.094		0.17
0.130	0.14	
0.084	1	
0.118	0.21	
0.136	0.19	
0.091		0.21
0.085		0.3
0.098		0.16
0.078		0.51
0.105	0.71	

Figure S10. a. XRD patterns during crystallization of aragonite from ACC with a Mg/Ca ratio of 0.09. **b.** Evolution of the peak area for the data shown in **a**. The crosses are the data; the blue line is the fit of a first order Avrami equation to this data using the Ezyfit toolbox of MATLAB. The table contains values for the rate of crystallization of aragonite from ACC samples of varying Mg/Ca ratios.

	Sample	Water Content n	H/D	Mg/Ca
Filtered Samples	H1	2.24	H	0.16
	H2	2.66	H	0.2
	H3	2.63	H	0.13
	HD1	3.46	0.5H/0.5D	0.17
	H4	2.52	H	0.17
	D1	3.3	D	0.2
	HD2	2.3	0.5H/0.5D	0.2
	H5	2.12	H	0.2
	HD3	3.15	0.25H/0.75D	0.2
	HD4	2.53	0.75H/0.25D	0.2
Freeze Dried Samples	H6	1.08	H	0.16
	D2	1.37	D	0.16
	H7	1.3	H	0.16
	D3	1.15	D	0.1
	H8	1.27	H	0.1
	D4	0.9	D	0.12
	H9	1.19	H	0.12

Table S1: Water content and Mg/Ca ratios determined for ACC samples prepared using the filtration and freeze-drying methods with H_2O, D_2O and H_2O/D_2O mixtures (amounts contained in the H/D column where H indicates pure H_2O, D indicates pure D_2O and other entries indicate the proportions of H_2O and D_2O) as solvent for a range of Mg/Ca ratios. The water content was determined using Thermogravimetric Analysis and the Mg/Ca ratios were determined using Laser-Ablation Inductively Coupled Plasma Mass Spectrometry.
